# The efficacy of pre-warming on reducing intraprocedural hypothermia in endovascular coiling of cerebral aneurysms

**DOI:** 10.1186/1471-2253-15-8

**Published:** 2015-01-21

**Authors:** Keun Man Shin, Jung Hwan Ahn, Il Seok Kim, Jong Young Lee, Sang Soo Kang, Sung Jun Hong, Hyun Mo Chung, Hee Jae Lee

**Affiliations:** Department of Anesthesiology and Pain Medicine, Kangdong Sacred Heart Hospital, Hallym University Medical Center, 150, Sungan-ro, Gangdong-gu, Seoul, 134-701 South Korea; Department of Emergency Medicine, Bundang Jesaeng General Hospital, Seongnam, South Korea; Department of Neurosurgery, Kangdong Sacred Heart Hospital, Hallym University Medical Center, Seoul, South Korea; Department of Pharmacology, School of Medicine, Kangwon National University, Chuncheon, South Korea

**Keywords:** Cerebral aneurysm, Hypothermia, Interventional neuroradiology, Pre-warming

## Abstract

**Background:**

The anesthetic management of patients undergoing endovascular treatment of cerebral aneurysms in the interventional neuroradiology suite can be challenged by hypothermia because of low ambient temperature for operating and maintaining its equipments. We evaluated the efficacy of skin surface warming prior to induction of anesthesia to prevent the decrease in core temperature and reduce the incidence of hypothermia.

**Methods:**

Seventy-two patients were randomized to pre-warmed and control group. The patients in pre-warmed group were warmed 30 minutes before induction with a forced-air warming blanket set at 38°C. Pre-induction tympanic temperature (Tpre) was measured using an infrared tympanic thermometer and core temperature was measured at the esophagus immediately after intubation (T0) and recorded at 20 minutes intervals (T20, T40, T60, T80, T100, and T120). The number of patients who became hypothermic at each time was recorded.

**Results:**

Tpre in the control and pre-warmed group were 36.4 ± 0.4°C and 36.6 ± 0.3°C, whereas T0 were 36.5 ± 0.4°C and 36.6 ± 0.2°C. Core temperatures in the pre-warmed group were significantly higher than the control group at T20, T40, T60, T80, T100, and T120 (P < 0.001). Compared to T0, core temperatures at each time were significantly lower in both two groups (P = 0.007 at T20 in pre-warmed group, P < 0.001 at the other times in both groups). The incidence of hypothermia was significantly lower in the pre-warmed group than the control group from T20 to T120 (P = 0.002 at T20, P < 0.001 at the other times).

**Conclusion:**

Pre-warming for 30 minutes at 38°C did not modify the trends of the temperature decrease seen in the INR suite. It just slightly elevated the beginning post intubation base temperature. The rate of decrease was similar from T20 to T120. However, pre-warming considerably reduced the risk of intraprocedural hypothermia.

**Trial registration:**

Clinical Research Information Service (CRiS) Identifier: KCT0001320. Registered December 19th, 2014.

## Background

The management of cerebral aneurysms has been conducted by surgical clipping, but recent advances in interventional neuroradiology (INR) have resulted in greater number of patients being managed with endovascular coiling in the INR suite [1, 2]. The anesthetic management of patients undergoing endovascular treatment of cerebral aneurysms in the INR suite can be challenged by intraprocedural hypothermia because of low ambient temperature for operating and maintaining its equipments. Endovascular procedures can be lengthy. Therefore, during prolonged procedures in the cold ambient temperature, the patients are more susceptible to hypothermia, especially the elderly and those with combined major medical comorbidities.

Although hypothermia has been used as a method for cerebral protection in the management of cerebrovascular procedures in aneurysms for many decades, a multicenter trial of mild hypothermia in relatively good-grade patients undergoing aneurysm surgery revealed no improvement in neurologic outcome [3, 4]. In general, hypothermia is associated with several adverse events including an increased susceptibility to cardiac dysrhythmias, delayed recovery from anesthesia, postoperative shivering, wound infections, and coagulopathies [5–9].

Patients undergoing endovascular procedures range from healthy patients with incidentally discovered unruptured aneurysms to moribund patients with devastating aneurysm ruptures. In good-grade patients, early assessment for neurologic deficits after coiling may be required and a smooth recovery from anesthesia is desirable for prevention of shivering and hypertension. If hypothermia occurs, there is not sufficient time to rewarm adequately because the endovascular procedure may finish very abruptly and considerable time is needed for the heat applied to the skin surface to extend into the core thermal compartment. Therefore, prevention of hypothermia and maintaining the body temperature near normal are recommended during endovascular coiling.

The objective of this study was to evaluate the efficacy of skin surface warming using a forced air warming blanket for 30 minutes prior to induction of anesthesia to prevent the decrease in core temperature that occurs during endovascular coiling of cerebral aneurysms and reduce the incidence of hypothermia.

## Methods

This consecutive, prospective randomized study was conducted after obtaining the approval of the Institutional Review Board (IRB No. 12-2-037) of Kang-Dong Sacred Heart Hospital (Seoul, South Korea). Written informed consent was obtained from either the patients or their families.

The enrolled patients were aged 20 to 80 years and were undergoing elective or emergency endovascular coiling to treat cerebral aneurysms with general anesthesia in the INR suite. Exclusion criteria included: a history of current infection, the intake of antipyretics within 24 hours before induction of anesthesia, a body mass index (BMI) exceeding 35 kg/m^2^, preoperative body temperature of more than 37.2°C before transfer to the INR suite, and patients with severe neurosurgical conditions whose treatment should not be delayed for the period required establishing skin surface warming. Patients who did not give consent for the pre-warming were also excluded.

Patients were randomly allocated to either the pre-warmed group or the control group with a sealed envelope method. In the pre-warmed group, patients were warmed for 30 minutes with a forced air warming blanket (3M™ Bair Hugger™ Full Body Blanket Model 300, Arizant Healthcare Inc., A 3 M Company, Eden Prairie, MN, USA) which covered the entire body except the head and neck, along with a blower (3M™ Bair Hugge™ Temperature Management Unit Model 505, Arizant Healthcare Inc., A 3 M company, Eden Prairie, MN, USA) set to medium level (38°C). Pre-warming was started before entering the INR suite and maintained until induction of anesthesia. In the control group, patients were covered only with two layers of cotton blanket that were not warmed before transfer to the INR suite and this was continued during the positioning. The forced air warming blanket in the pre-warmed group and the cotton blanket in the control group were removed at induction of anesthesia. An upper body blanket (3M™ Bair Hugger™ Upper Body Blanket Model 522, Arizant Healthcare Inc., A 3 M Company, Eden Prairie, MN, USA) was applied to the patients in both groups. The forced air warming device was operated if the core temperature of patients decreased below 35.5°C during endovascular procedure. The temperature output of the blower was set at high level (43°C).

In all patients, glycopyrrolate 0.2 mg was administered intramuscularly for premedication. When the patients arrived in the INR suite, they were all monitored with standard equipments including an electrocardiogram, non-invasive blood pressure cuff, pulse oximetry, and capnography. The INR room temperature was measured with a digital thermometer (YT302, UINS, Seoul, Korea) placed near the patient but away from the heat-generating equipment and preserved at 19 to 21°C. Pre-induction tympanic temperature (Tpre) was measured with an infrared tympanic thermometer (Instant Thermometer HM3, Braun, San Diego, USA) before induction of anesthesia and the high reading from three consecutive measurements was recorded. Anesthesia was induced and maintained with continuous infusion of propofol and remifentanil using a target control infusion pump (Orchestra® Base Primea, Fresenius Vial, Brezins, France). Rocuronium 0.7-0.8 mg/kg was administered intravenously to facilitate tracheal intubation. The radial artery cannulation was done with a 22-gauge angiocatheter for continuous arterial pressure monitoring to avoid blood pressure surges during intubation.

An esophageal temperature probe (DeRoyal Esophageal Stethoscope with Temperature Sensor, DeRoyal Industries Inc., Powell, TN, USA) was inserted 25 cm into the esophagus under direct vision for measuring the core temperature. The core temperature was monitored and recorded electronically using Datex-Ohmeda monitor system (Datex-Ohmeda S/5™ Compact Anesthesia Monitor, GE Healthcare, Helsinki, Finland). The core temperature was measured immediately after intubation (T0) and recorded at 20 minute intervals (T20, T40, T60, T80, T100, and T120) until the completion of endovascular procedures. Hypothermia defined as a core temperature less than 36.0°C in according to the current guideline [10]. The number of patients who became hypothermic at each time was recorded.

Mechanical ventilation was controlled to maintain an end-tidal carbon dioxide tension between 28 and 32 mmHg with a tidal volume of 8–10 ml/kg and a respiratory rate of 8–12 breaths/min under 40-60% oxygen-air mixture according to the patients. Central venous catheterization was performed in the right infraclavicular axillary vein by real-time guidance of ultrasonography. Intravenous fluids that had been stored at 37°C were infused via central line intraoperatively. Urinary catheterization was performed to measure urine output in order to manage diuresis secondary to administration of mannitol and radiographic contrast. Hemodynamic variables were adjusted to the preoperative value within 20% through support of the circulation with fluids and injection of low doses of phenylephrine, dopamine, esmolol, or nicardipine intermittently when clinically indicated. Procedure-related complications were bounded as an intraprocedural aneurysm perforation, thromboembolic complications, or any other complications associated with the endovascular procedure and recorded.

In patients with unruptured aneurysm being secured completely, extubation was done for neurologic assessment. In recovery, shivering was graded by an investigator blinded to core temperature using a 4 point scale (0 = none, 1 = mild and intermittent shivering, 2 = moderate shivering, 3 = intense and continuous shivering). If a coiling was complicated or the recovery was expected to be delayed, the patient was sedated and transferred to the neurosurgical intensive care unit.

Sample size calculation was based on the results of previous research [11]. We tested the null hypothesis that there is no difference in core temperature between the pre-warmed and control group. We wanted to detect a difference of means of 0.3°C and assumed that standard deviation (SD) for core temperature was 0.6°C. For each group, 36 subjects were required to reject the null hypothesis with 80% power and a significant level of 0.05.

Data collection included patient demographics, Tpre, core temperature at each time, the number of patients with hypothermia at each time, location and size of aneurysm, amount of subarachnoid hemorrhage (SAH) using Fisher grade [12], procedure-related complications, and the number of patients with shivering in recovery. Data were analyzed by SPSS 19.0 (IBM Inc., Armonk, NY, USA). Comparisons of core temperature at each time between the groups were performed using an independent *T* test. Comparisons of core temperature between T0 and other times (T20-120) within each group were made using paired T tests. The number of patients with hypothermia (<36.0°C) at each time, procedure-related complications and shivering between the groups were compared using a chi-square or Fisher’s exact test. Other categorical variable between the groups were compared using a chi-square or Fisher’s exact test as appropriate. To compare other continuous variable between the groups, an independent *T* test or Mann–Whitney *U* test was used where appropriate. Data were expressed as mean ± SD or number of patients. P values < 0.05 were considered statistically significant.

## Results

**Figure 1:**
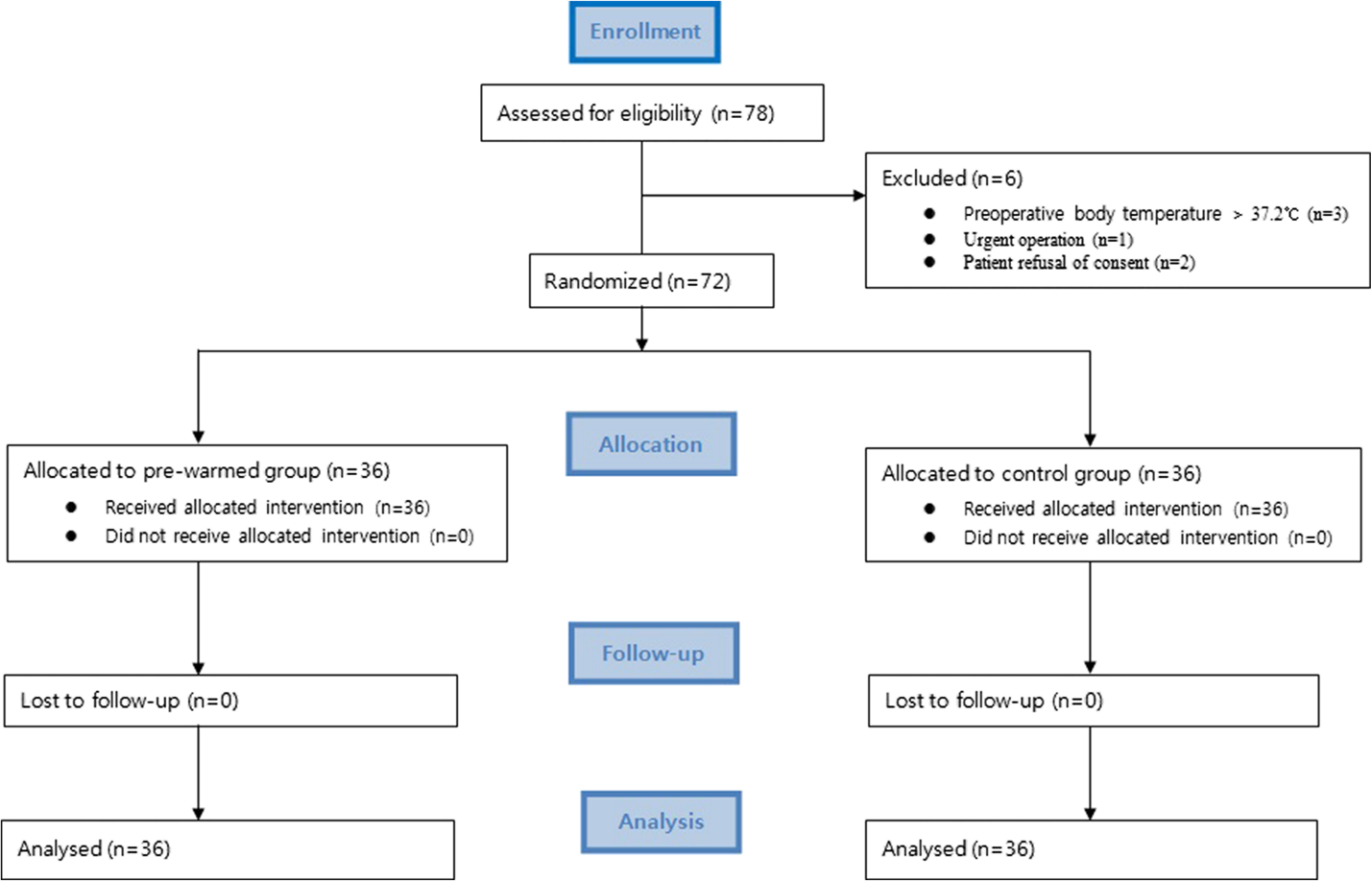
**Consort flow diagram of the patients.**

A total of 78 patients undergoing endovascular coiling of cerebral aneurysms were assessed for eligibility from October 22, 2012 to December 31, 2013. Of these, 6 patients were excluded because of preoperative body temperature of more than 37.2°C before transfer to the INR suite in 3 patients, urgent operation in 1 patient, and refusal of the consent in 2 patients. Finally, 72 patients were enrolled in this study. In the final analysis, the pre-warmed group consisted of 36 patients and the control group of 36 patients (Figure 1).

Demographic characteristics of patients and periprocedural variables are shown in Table 1. Age, gender, BMI, and number of ruptured and unruptured aneurysm were comparable between the two groups. Duration of anesthesia and operation and INR room temperature did not statistically differ. Tpre measured using an infrared tympanic thermometer in the control and pre-warmed group were 36.4 ± 0.4°C and 36.6 ± 0.3°C, respectively. Location and size of aneurysm and Fisher grade were comparable between the groups.

**Figure 2:**
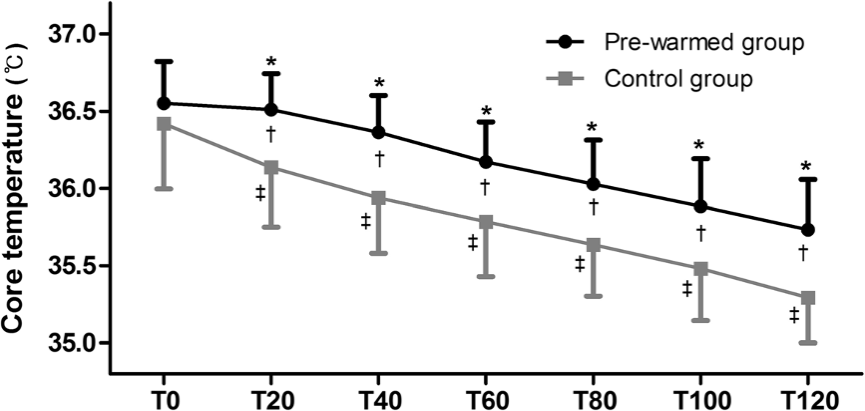
**Core temperature according to times (°C).** Results are presented as means ± SD. *Indicates statistically significant difference between the groups at each time. † indicates statistically significant difference between T0 and other times in the pre-warmed group. ‡ indicates statistically significant difference between T0 and other times in the control group. T0: 0 minute after intubation, T20: 20 minutes after intubation, T40: 40 minutes after intubation, T60: 60 minutes after intubation, T80: 80 minutes after intubation, T100: 100 minutes after intubation, T120: 120 minutes after intubation.

The changes in core temperature according to times are shown in Figure 2. T0 in the control group and pre-warmed group were 36.5 ± 0.4°C and 36.6 ± 0.2°C, respectively. There was no statistically significant difference at T0 between the groups. Core temperatures in the pre-warmed group were significantly higher than those of the control group at T20, T40, T60, T80, T100, and T120 (P < 0.001 at all times). Compared to T0, core temperatures at T20, T40, T60, T80, T100, and T120 were all significantly lower in both two groups (P = 0.007 at T20 in pre-warmed group, P < 0.001 at the other all times in both groups). The highest difference in core temperature between the groups was 0.5°C at T40. The difference in core temperature between the groups was identified as 0.4°C at the other times except T0 which was 0.1°C. The mean core temperatures in the pre-warmed group were maintained above 36°C until T80.

**Figure 3:**
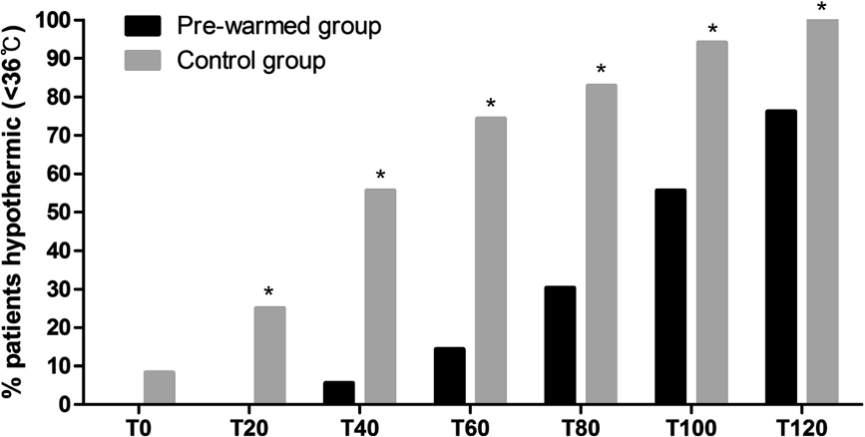
**Percentage of patients who were hypothermic (<36.0°C** **) at each time in each group.** *Indicates statistically significant difference between the groups at each time.

Figure 3 shows the percentage of patients who were hypothermic (less than 36.0°C) at each time in each group. Three patients in the control group were hypothermic at T0 (8.3%). The pre-warmed group/control group incidence (percentage) of hypothermia at each time was 0 (0%)/9 (25%) at T20, 2 (5.6%)/20 (55.6%) at T40, 5 (14.3%)/26 (74.3%) at T60, 10 (30.3%)/29 (82.9%) at T80, 15 (44.4%)/32 (94.1%) at T100, and 16 (76.2%)/29 (100%) at T120. The incidence of hypothermia was statistically significant lower in the pre-warmed group than the control group at T20, T40, T60, T80, T100, and T120 (P = 0.002 at T20, P < 0.001 at the other all times).

**Table 1:** **Demographic characteristics**

	Pre-warmed group	Control group	P value
	(n = 36)	(n = 36)	
Age (years)	56 ± 15	60 ± 13	0.279
Gender (male:female)	10:26	14:22	0.317
Body mass index (kg/m^2^)	23.4 ± 3.1	23.8 ± 3.4	0.536
Aneurysm			0.165
Ruptured	25	30	
Unruptured	11	6	
Duration of anesthesia (min)	137 ± 50	139 ± 40	0.680
Duration of operation (min)	100 ± 45	100 ± 37	0.800
INR room temperature (°C)	20.0 ± 0.5	19.8 ± 0.6	0.391
Tpre (°C)	36.6 ± 0.3	36.4 ± 0.4	0.114

Values are mean ± SD or number of patients.

Tpre: Pre-induction tympanic temperature.

Procedure-related complications and shivering are shown in Table 2. There is no significant difference in procedure-related complications between the groups. Six patients of the pre-warmed group and 9 patients of the control group were excluded from analysis on shivering because these patients were sedated with an endotracheal tube in situ and transported directly to the neurosurgical intensive care unit for ventilation and delayed awakening. In the recovery room, shivering was observed in 3 out of 30 patients of the pre-warmed group (10.0%) and 6 out of 27 patients of the control group (22.2%). There was no statistically significant difference between the groups (P = 0.283). In grading of shivering, 3 patients in the pre-warmed group were grade 1, whereas 4 patients as grade 1 and 2 patients as grade 2 in the control group.

**Table 2:** **Procedure-related complications and shivering**

	Pre-warmed group (n = 36)	Control group (n = 36)	P value
Procedure-related complication*			0.840
IAP	1 (2.8)	1 (2.8)	
Thromboembolic complication	1 (2.8)	2 (5.6)	
Shivering (presence: absence)^†^	3 (10.0): 27 (90.0)	6 (22.2): 21 (77.8)	0.283

Values are number of patients (%).

IPA: intraprocedural aneurysmal perforation.

*Indicates Procedure-related complication which is observed in 36 patients of both groups.

^†^Indicates shivering which is observed in 30 patients of the pre-warmed group and 27 patients of the control group.

## Discussion

In this study, we found that pre-warming for 30 minutes at 38°C did not modify the trends of the temperature decrease seen in the INR suite. Although the difference in initial core temperature was small, core temperature at 20 minutes after intubation of the control group decreased significantly in contrast with the pre-warmed group and the rate of decrease is similar such that the difference in core temperature 40 minutes after intubation was 0.5°C and the difference between the two groups at 120 minutes was 0.4°C. Pre-warming did not affect this decrease. It just slightly elevated the beginning post intubation base temperature by 0.1°C. However, with respect to the incidence of hypothermia, pre-warming considerably reduced the risk of intraprocedural hypothermia. The majority of the pre-warmed group was normothermic or mildly hypothermic in contrast to the control group by the end of the procedure.

Intraoperative hypothermia represents a three-phase pattern [13]. An initial rapid decrease in core temperature observed during the first hour of general anesthesia is related to the anesthetic-induced impairment of centrally mediated thermoregulatory control leading to the inhibition of thermoregulatory vasoconstriction and mainly the core-to-peripheral redistribution of heat, which is due to peripheral vasodilatation by anesthetic drugs. A relatively slow, linear decrease follows subsequent 2 or 3 hours. It results from the thermal imbalance that heat loss exceeds metabolic heat production. Heat loss to the environment is mediated by four heat transfer pathways, radiation, convection, conduction, and evaporation. Finally, core temperature reaches a plateau phase that remains constant, even during prolonged surgery.

Core temperature usually decreases 1 to 1.5°C during the initial redistribution phase [14]. Because flow of heat requires a temperature gradient between the core and peripheral thermal compartments, the degree of redistribution hypothermia depends on the patient’s initial body heat content. Skin surface warming before induction of anesthesia increases heat contents of the peripheral compartment and decreases the core-to-peripheral temperature gradient, which reduces the core-to-peripheral flow of heat [15]. Such pre-warming is accepted as the effective technique to reduce redistribution hypothermia. In addition, many patients like the warming, especially when they are in a cool environment with only a surgical gown on. Heat loss from the body to a cold environment aggravates intraoperative hypothermia. Approximately 90% of the heat loss is through the skin surface, with radiation and convection usually contributing far more to the process than evaporation or conduction [16]. Therefore, the skin is the site of focus in modulating thermal manipulation.

Previous studies have shown that pre-warming to prevent hypothermia needed to be performed for at least 1 hour [17–19]. However, such prolonged pre-warming is difficult during clinical practice and emergency situations because it is time consuming. Horn et al. performed a short time periods of pre-warming study and found that pre-warming of only 10 or 20 minutes at 44°C reduced considerably the risk of perioperative hypothermia and postoperative shivering [20]. However, they measured core temperature at the tympanic membrane using a tympanic membrane sensor. Mean body temperature (MBT) is calculated from core (Tcore) and mean skin temperature (Tskin) using the following formula: MBT = 0.64 × Tcore + Tskin [21]. Core temperature will not change in spite of pre-warming because of the sweating mechanism [15]. In our study, pre-induction core temperature was difficult to obtain because of invasiveness of esophageal temperature probe insertion in awaked patients and skin temperature was not measured for misinterpretation of MBT which was influenced by skin temperature undergoing skin surface warming. There was a discrepancy between Tpre and T0 in our study. The reason is probably related to a warmed air indirectly affecting the tympanic membrane and an inaccuracy of the infrared tympanic thermometer. Thus, we measured the highest value of the tympanic temperature from consecutive three measurements to decrease error and considered the esophageal temperature as the core temperature.

Ideally the INR suite should be prepared for anesthetic care similarly to a standard operating room. However, the INR suite has not generally been designed with the needs of the anesthesiologist in mind and requires a low ambient temperature for operating and maintaining its equipment. Thus, for long periods of the procedure on a radiology table during endovascular coiling, the patients are more susceptible to hypothermia. In our study, three patients in the control group were already hypothermic at T0. Although the trend of decrease in core temperature during procedure was not enough to prevent completely, 30 minutes of active skin-surface pre-warming at 38°C increased the body heat content before induction and initial core temperature slightly and reduced the incidence of hypothermia related to redistribution. Exposure to a warm environment for 30 minutes did influence the heat content of the peripheral compartment and reduce the core-to-peripheral flow of heat. In previous study [20], more short duration of pre-warming at high temperature could reduce the incidence of hypothermia. In our study, we conducted pre-warming at medium temperature setting in considering of burns from higher temperature setting. However, this hot air warming blanket should not produce burns when used appropriately. Even if we conducted intraprocedural warming with upper body blanket set at 43°C once the core temperature decreased below 35.5°C, hypothermia was difficult to reverse because large amount of heat is needed for applying the skin surface to extend into the core thermal compartment. An additional intraprocedural use of a warming device such as under body warming blanket may have prevented the heat loss through radiation and convection.

Various factors such as core hypothermia, pain, and high doses of remifentanil were associated with postoperative shivering [22]. Endovascular procedures did not produce severe pain and did not require high doses of remifentanil. Therefore, shivering was mainly affected by core hypothermia. In our study, there was a confounding factor for evaluation of shivering because some of patients were excluded from analysis according to be transported the neurosurgical intensive care unit under sedation for delayed awakening in more complicated cases. Except for these, occurrence of shivering was not statistically different between the groups. However, the incidence of grade 2 meaning moderate shivering was observed in the control group, whereas none in the pre-warmed group. The number of patients included is too low for interpretation. Large scale trial is required to evaluate whether there are the beneficial effects of pre-warming on the shivering.

Several studies on prevention of hypothermia with pre-warming have shown mixed results depending on duration of pre-warming and combination with intraoperative warming [17–20]. In our study, we conducted intraprocedural warming with upper body blanket in all patients of both groups if the core temperature decreased to 35.5°C so as to exclude the effect of active warming during the procedure and detect the effect of pre-warming on core temperature in the intraprocedural periods. 30 minutes of pre-warming did not prevent the trends of decrease of core temperature from heat loss to cold environment in the INR suite. It only increased the initial body heat content and prevented heat loss from redistribution in initial short periods. However, in aspects of prevention of hypothermia, it reduced the incidence of hypothermia.

## Conclusion

30 minutes of pre-warming at 38°C before induction of anesthesia was not enough to prevent the decrease in core temperature but reduced the incidence of hypothermia. In patients undergoing coiling of aneurysm at risk of hypothermia in a cold environment, pre-warming should be considered as part of the anesthetic management. A decrease in post-induction core temperature is still unavoidable, but the unintentional intraoperative hypothermia in patients undergoing endovascular procedures in the INR suite is largely preventable.

## Abbreviations

INR: Interventional neuroradiology
BMI: Body mass index
SAH: Subarachnoid hemorrhage
MBT: Mean body temperature
Tcore: Core temperature
Tskin: Skin temperature.
